# Health‐related quality of life and patient‐reported outcome measures in economic evaluation of urological technologies: challenges and opportunities

**DOI:** 10.1111/bju.70365

**Published:** 2026-06-30

**Authors:** Mohammed El Bahnasawi, Charlotte Moss, Netty Kinsella, Kathie Alexina Wong, Mieke Van Hemelrijck

**Affiliations:** ^1^ Urology Department, Freeman Hospital Newcastle University Teaching Hospitals Newcastle Upon Tyne UK; ^2^ North Cumbria Integrated Care Trust Carlisle UK; ^3^ King's College London Faculty of Life Sciences and Medicine, TOUR London UK; ^4^ Urology Department Royal Marsden Hospital London UK

**Keywords:** health economics, cost‐utility analysis, urology, quality‐adjusted life year (QALY), patient‐reported outcomes, health‐related quality of life

## Abstract

**Objective:**

To examine the role of health‐related quality of life (HRQoL) assessments and patient‐reported outcome measures (PROMs) in the economic evaluation of urological interventions, highlighting methodological challenges, and identifying areas for future development.

**Methods:**

This narrative review synthesises key frameworks for economic evaluation in urology, describes methods for utility derivation, and critically appraises the application of both generic and disease‐specific PROMs. Evidence is drawn from major urological trials and model‐based evaluations, with attention to their impact on health technology assessment decisions.

**Results:**

Patient‐reported outcome measures underpin cost‐utility analyses across the full spectrum of urological conditions, including benign prostatic hyperplasia (BPH), urinary incontinence, overactive bladder, bladder cancer, renal cancer, and prostate cancer. Generic instruments such as the EuroQoL five Dimensions (EQ‐5D) and 36‐item Short‐Form Health Survey (SF‐36) facilitate cross‐disease comparison but may lack sensitivity to urological symptoms. Disease‐specific tools (e.g., International Prostate Symptom Score, Overactive Bladder Questionnaire, 26‐item Expanded Prostate cancer Index Composite) provide granularity but often require mapping to utility values, introducing uncertainty. Trial‐based evaluations (e.g., Trans European Research into the use of Management Policies for LUTS suggestive of BPH in Primary Healthcare [TRIUMPH], Single‐Incision Mini‐Slings [SIMS], Refractory Overactive Bladder: Sacral NEuromodulation vs BoTulinum Toxin Assessment [ROSETTA]) demonstrate that PROM‐derived quality‐adjusted life year differences can decisively influence cost‐effectiveness. Surgery‐related PROMs including the Consultation and Relational Empathy measure can capture important short‐term recovery outcomes not measured by generic tools.

**Conclusions:**

Health‐related quality of life and PROMs are foundational to value‐based urological care. Accurate and context‐sensitive measurement ensures that cost‐utility analyses reflect patient experience as well as clinical outcomes. Future priorities include development of preference‐based urology‐specific PROMs, improved digital/electronic PROM capture, validation of mapping algorithms, and integration of surgery‐related PROMs into economic evaluations.

**Abbreviations**
CARE: Consultation and Relational Empathy (measure)
CEA: cost‐effectiveness analysis
CUA: cost‐utility analysis
DCE: discrete choice experiment
EORTC (QLQ‐C30) (QLU‐C10D) (QLQ‐PR25): European Organisation for the Research and Treatment of Cancer (Quality Of Life Questionnaire – 30‐item core) (Quality of Life Utility – Core 10 Dimensions) (Quality Of Life Questionnaire – prostate specific 25‐item)
EPIC(‐26): Expanded Prostate Cancer Index Composite (26‐items)
EQ‐5D: EuroQoL five Dimensions
FACT‐P: Functional Assessment of Cancer Therapy – Prostate
HRQoL: health‐related quality of life
HTA: health technology assessment
HUI‐3: Health Utilities Index Mark 3
ICER: incremental cost‐effectiveness ratio
ICIQ: International Consultation on Incontinence Questionnaire
MAUT: multi‐attribute utility theory
MID: minimally important difference
NICE: National Institute for Health and Care Excellence
OAB‐q: Overactive Bladder Questionnaire
PBM: preference‐based measure
(e)PROM: (electronic) patient‐reported outcome measure
QALY: quality‐adjusted life‐year
SF‐6D: Short‐Form six Dimensions

## Introduction

Over recent decades, health‐related quality of life (HRQoL) has emerged as a critical outcome in urology, reflecting a broader paradigm shift away from focusing solely on clinical endpoints to incorporating patient perspectives on their own wellbeing, functioning, and symptomology [1]. Urological conditions such as prostate cancer, LUTS, urinary incontinence, and sexual dysfunction can have profound and long‐lasting effects on physical, psychological, and social domains of health [2, 3, 4]. Measuring these effects allows healthcare providers and policymakers to assess the value of interventions beyond survival, capturing quality of life following treatment [5].

The importance of measuring HRQoL has become increasingly recognised alongside the emergence of value‐based healthcare and health technology assessment (HTA) frameworks, where decisions about treatment are increasingly guided by cost‐effectiveness evidence [6, 7]. To inform such evaluations, HRQoL data are converted into preference‐based utility values, which quantify health states on a scale anchored between 0 (equivalent to death) and 1 (perfect health) [8]. These utilities are then combined with survival data to generate quality‐adjusted life‐years (QALYs) – a measure that captures both the length and quality of life gained from an intervention [9].

Despite advances in HRQoL measurement across many healthcare disciplines, progress in urology has been more limited. Previously, disease‐specific patient‐reported outcome measures (PROMs) such as the IPSS, Overactive Bladder Questionnaire (OAB‐q), and Expanded Prostate Cancer Index Composite (EPIC) have been relied upon to provide symptom and functional information but are not preference based [10, 11, 12]. These measures therefore cannot directly generate the utility values required for cost‐utility analyses. In contrast, generic preference‐based instruments, such as the EuroQoL five Dimensions (EQ‐5D) or Short‐Form six Dimensions (SF‐6D), enable comparison across diseases but lack sensitivity to urology‐specific domains such as urinary continence, sexual functioning, and body image [8, 13]. Surgery‐related PROMs, such as the Consultation and Relational Empathy (CARE) measure, offer additional value in capturing recovery trajectories and the quality of the patient‐clinician interaction following urological procedures, yet are rarely integrated into economic evaluations.

One advance has been the development of the European Organisation for the Research and Treatment of Cancer Quality of Life Utility – Core 10 Dimensions (EORTC QLU‐C10D), a cancer‐specific preference‐based measure (PBM) derived from the EORTC quality of life questionnaire – 30‐item core (EORTC QLQ‐C30), which highlights the potential of adapting existing instruments into utility‐generating tools [14]. Given this evolving landscape, the present review aimed to provide a narrative synthesis of current approaches to HRQoL and PBMs in urology. It presents the main types of economic evaluation, reviews the role of generic, cancer‐specific, disease‐specific, and surgery‐related PROMs, and discusses methodological challenges and priorities for future research.

## Economic Evaluation Frameworks

Economic evaluation provides a methodology for comparing alternate healthcare interventions in terms of their costs and the outcomes they achieve [15]. In this era of finite healthcare resources, these evaluations underpin decisions about which treatments provide sufficient value to justify their adoption. Understanding different evaluation frameworks is essential for clinicians engaged in research or interpreting HTA guidance.

The fundamental distinction between economic evaluation types lies in how outcomes are measured and valued [15]. Table 1 below provides a summary overview; the paragraphs that follow describe each in more detail.

**Table 1:** Summary of economic evaluation methods used in healthcare.

Method	How outcomes are measured	Main strength	Key limitation
Cost‐minimisation (CMA)	Costs only (outcomes assumed equivalent)	Simple; identifies cheapest option	Requires proven equivalence; rarely applicable
Cost‐effectiveness (CEA)	Natural clinical units (e.g., symptom episodes avoided, life‐years gained)	Clinically intuitive; useful within disease area	Cannot compare across different conditions
Cost–benefit (CBA)	Monetary value of health outcomes	Enables comparison with non‐health investments	Ethically and methodologically complex
Cost‐utility (CUA)	QALYs (utility × time)	Cross‐disease comparison; required by NICE	Depends critically on accuracy of utility values

NICE, National Institute for Health and Care Excellence.

Cost‐minimisation analysis is the simplest form and is only appropriate when interventions have been proven to yield equivalent clinical outcomes [16]. Cost‐effectiveness analysis (CEA) extends this by comparing costs with outcomes expressed in a natural unit – e.g., symptom improvement or life‐years gained [16]. CEA is useful within a single disease area but cannot enable cross‐condition comparisons. Cost–benefit analysis attempts to overcome this by expressing both costs and benefits in monetary terms, although this raises profound ethical challenges when applied to health [16].

The most widely adopted approach in healthcare is cost‐utility analysis (CUA), which measures outcomes in terms of QALYs [17]. A QALY integrates both the quantity and quality of life by multiplying the utility value of a health state by the time spent in it. By converting health outcomes into a single utility index (0 = death, 1 = perfect health), CUA enables comparison of diverse interventions on a common scale. Agencies such as the National Institute for Health and Care Excellence (NICE) require CUA for most HTA submissions, with cost‐effectiveness thresholds typically set at £20 000–30 000/QALY [18]. Consequently, even small variations in utility values can alter cost‐effectiveness conclusions – underscoring the critical importance of accurate HRQoL measurement in urology.

## Categories of PROMs Used in Urology

The selection of appropriate instruments to measure HRQoL is a critical component of conducting high‐quality economic evaluations. In urology, where conditions often exert complex and multidimensional effects on physical and psychosocial wellbeing, this choice is particularly consequential. PROMs used in urology span four main categories: (i) generic PBMs, (ii) cancer‐specific PBMs, (iii) urology disease‐specific measures, and (iv) surgery‐related measures. Each serves a distinct purpose and carries inherent strengths and limitations (Tables 2 and 3).

**Table 2:** Categories of HRQoL measures used in urology.

PROM category	Examples	Domains captured	Utility generation	Urological application
General/generic preference based	EQ‐5D‐3L, EQ‐5D‐5L, SF‐6D, HUI‐3	Mobility, self‐care, usual activities, pain, anxiety	Direct – utility tariff applied	Cost‐utility analyses; HTA submissions (NICE); cross‐disease comparison
Cancer‐specific preference based	EORTC QLU‐C10D, FACT‐G	Physical, role, social, emotional functioning; fatigue, pain, nausea	Direct (EORTC QLU‐C10D) or mapping (FACT‐G)	Prostate, bladder, and kidney cancer trials; EORTC‐based studies
Urology disease specific	IPSS, OAB‐q, ICIQ, EPIC‐26, FACT‐P, EORTC QLQ‐PR25, BCI, FKSI‐19, FACT‐VCI	Urinary, sexual, bowel function; disease‐specific symptoms	Requires mapping to EQ‐5D or utility instrument	BPH, LUTS, prostate cancer, bladder cancer, urinary incontinence, renal cancer
Surgery‐related	CARE, PROM‐based recovery scales	Recovery experience, relational empathy, postoperative status	Generally not preference‐based; used alongside generic measures	Prostatectomy, cystectomy, minimally invasive urological procedures

BCI, Bladder Cancer Index; CARE, Consultation and Relational Empathy; DCE, discrete choice experiment; EQ‐5D(‐3L)(‐5L), EuroQoL five Dimensions (three Levels) (five Levels); FACT(‐G)(‐VCI), Functional Assessment of Cancer Therapy (– General) (– Vanderbilt Cystectomy Index); FKSI‐19, Functional Assessment of Cancer Therapy‐Kidney Symptom Index; HUI‐3, Health Utilities Index Mark 3; SF‐6D, Short‐Form six Dimensions.

**Table 3:** Strengths and limitations of PROM categories for economic evaluation.

PROM category	Utility for CUA	Limitations	Role in economic evaluation
Generic preference based (e.g., EQ‐5D)	Direct – tariff‐derived utility	Limited responsiveness to urological symptoms (continence, sexual function, body image); may miss clinically meaningful changes; ceiling effects in milder conditions	‘Gold standard’ for HTA; enables cross‐disease QALY comparison
Cancer‐specific preference based (e.g., EORTC QLU‐C10D)	Direct (EORTC QLU‐C10D via DCE weights)	Cancer specific only; national value sets incomplete for some countries; not validated for benign urological conditions	Ideal for oncological urology where EORTC QLQ‐C30 is already collected
Urology disease specific (e.g., IPSS, EPIC‐26)	Mapping required	Cannot directly generate utilities; mapping introduces statistical uncertainty; domain mismatch reduces accuracy; validation varies by population	Captures clinical nuance; must be combined with generic instrument for economic evaluation
Surgery related (e.g., CARE)	Not preference based	Designed for recovery assessment, not utility generation; requires linkage to generic PROMs for economic analysis	Supplements short‐term postoperative outcomes; particularly valuable for prostatectomy and cystectomy evaluation

### Generic PBMs

Generic PBMs – including the EQ‐5D, SF‐6D, and Health Utilities Index Mark 3 (HUI‐3) – have been designed to provide a standardised method for describing and valuing health states across all diseases and populations [19]. They enable the calculation of utilities that are comparable across clinical areas, a key requirement for HTA and national reimbursement decision‐making.

The EQ‐5D, developed by the EuroQol Group, is the most widely used instrument in this category [8]. It comprises five dimensions: mobility, self‐care, usual activities, pain/discomfort, and anxiety/depression. Utility weights derived from general population surveys allow these health states to be converted into QALY estimates. Its conciseness and ease of use have made it the default instrument in many clinical trials and HTA submissions.

However, generic instruments such as the EQ‐5D are often criticised for limited sensitivity in conditions that affect domains not explicitly captured by their dimensions. This is particularly relevant in urology, where urinary function, sexual health, and body image are key but may not significantly alter EQ‐5D scores. For instance, patients recovering from radical prostatectomy may experience marked declines in continence and sexual function – domains underrepresented in generic utility measures. Consequently, clinically meaningful HRQoL improvements from urological interventions may appear less striking when evaluated with generic instruments alone.

Short‐term surgical outcomes represent a further limitation. Studies using the EQ‐5D or EORTC QLQ‐C30 in the immediate postoperative period following prostatectomy, cystectomy, or minimally invasive procedures often fail to detect recovery trajectories that are clinically relevant. This is because the dimensions of generic instruments are broadly framed and may be relatively insensitive to the transient but important changes that occur in the weeks following surgery.

### Cancer‐Specific PBMs

Cancer‐specific PBMs, most notably the EORTC QLU‐C10D, have been developed to address the responsiveness limitations of generic instruments within oncology settings. These tools retain sensitivity to cancer‐specific symptoms and treatment effects while producing utility values compatible with cost‐utility analysis – a dual capability that generic instruments often cannot fully provide in oncological urology.

### Urology Disease‐Specific PROMs

Disease‐specific instruments have been developed to capture the aspects of health most relevant to a particular condition. In urology, widely used examples include the IPSS for LUTS, the OAB‐q, the International Consultation on Incontinence Questionnaire (ICIQ) for incontinence, the 26‐item EPIC (EPIC‐26) for prostate cancer, the Functional Assessment of Cancer Therapy – Prostate (FACT‐P) for prostate cancer‐specific wellbeing, the FACT – Vanderbilt Cystectomy Index (FACT‐VCI) for urinary symptoms in cancer, and the Bladder Cancer Index (BCI) for bladder cancer [10, 11, 12]. These instruments provide detailed assessment of symptoms, functional status, and treatment‐related toxicity.

Although disease‐specific PROMs are more responsive to clinical change, they cannot generate utility values for cost‐utility analysis without mapping. Mapping uses statistical models to estimate utility values from disease‐specific scores by linking them to responses from a generic PBM such as the EQ‐5D [20]. While mapping can extend the usability of existing data, it introduces additional uncertainty that can substantially alter cost‐effectiveness conclusions (see Section on Methodological Considerations).

### Surgery‐Related PROMs: The CARE Instrument

Surgery‐related PROMs represent an important and often overlooked category in economic evaluations of urological procedures. The CARE measure was originally developed to assess the quality of the clinical consultation and the degree to which patients feel heard, respected, and supported during clinical encounters. However, in the context of urological surgery it has been adapted and used more broadly to capture recovery experience, relational empathy during perioperative care, and the quality of postoperative support.

For procedures such as radical prostatectomy, cystectomy, and minimally invasive urological surgeries, CARE provides granularity on recovery trajectories that generic instruments fail to detect. Specifically, it captures the experience of care delivery – an aspect of value that contributes to patient‐reported wellbeing but is not reflected in utility scores or symptom‐based PROMs. When integrated alongside a generic PBM, CARE can provide a more complete picture of the patient journey, and the combined approach can strengthen HTA submissions by demonstrating the breadth of value delivered by surgical interventions.

From an economic evaluation perspective, CARE is not preference based and cannot independently generate utility values. However, its use alongside EQ‐5D or EORTC QLU‐C10D can provide complementary evidence about the patient experience of care, supporting narrative submissions to commissioners and informing shared decision‐making in surgical urology.

### Balancing PROM Categories in Practice

The optimal strategy in urological research is to combine generic and disease‐specific instruments within clinical trials, with consideration of surgery‐related tools where relevant. The generic PBM provides the utility data required for cost‐effectiveness analysis; the disease‐specific PROM ensures sensitivity to clinical change; and surgery‐related PROMs capture important perioperative and recovery dimensions. When used together, they allow both policy‐level and patient‐level outcomes to be comprehensively captured.

## The EORTC QLU‐C10D

The development of the EORTC QLU‐C10D represents a major advancement in preference‐based health measurement in oncology. Developed from the EORTC QLQ‐C30, it enables the calculation of QALYs while retaining sensitivity to cancer‐specific symptoms and treatment effects, thereby bridging the gap between clinical relevance and economic utility.

Created by the EORTC Quality of Life Group using multi‐attribute utility theory (MAUT), the EORTC QLU‐C10D captures 10 key domains from the EORTC QLQ‐C30: physical, role, social, and emotional functioning, as well as pain, fatigue, sleep, appetite loss, nausea, and bowel problems [14]. Utility weights were established through discrete choice experiments (DCEs) with population samples across Australia, the UK, Germany, the Netherlands, and France, with work ongoing in Asia and North America. Severity levels are derived directly from EORTC QLQ‐C30 responses, ensuring conceptual continuity and minimising respondent burden.

Validation studies have demonstrated that the EORTC QLU‐C10D has acceptable psychometric properties and greater sensitivity to cancer‐related changes than generic instruments like the EQ‐5D [21, 22]. It is particularly responsive to symptoms such as fatigue, nausea, and emotional distress – symptoms often underrepresented in generic measures. This is especially relevant in prostate cancer settings, where treatment effects including bowel or sexual dysfunction may not be accurately assessed using broader tools.

In prostate cancer, where the EORTC QLQ‐C30 and its disease‐specific module the EORTC QLQ – prostate specific 25‐item (QLQ‐PR25) are frequently administered, the EORTC QLU‐C10D can be directly derived from existing trial data without additional respondent burden. This makes it a practical and methodologically coherent choice for generating utilities in prostate cancer cost‐utility analyses. Its use is increasingly recommended when the QLQ‐C30 is part of trial data collection.

Existing limitations include the fact that the EORTC QLU‐C10D is cancer‐specific and therefore may not capture domains relevant to non‐oncological urological conditions, such as urinary frequency, urgency, or incontinence. Furthermore, while national value sets are expanding across more countries, differences in how health states are valued between populations introduce additional uncertainty in international economic evaluations.

The development of the EORTC QLU‐C10D illustrates how a PBM can evolve from established, non‐preference‐based PROMs. For urology researchers, it provides a replicable template: instruments such as the EPIC‐26 or ICIQ could, in principle, be adapted into preference‐based formats using MAUT and DCE methodology. Table 4 provides a step‐by‐step worked example of how EORTC QLQ‐C30 data collected in a prostate cancer randomised controlled trial can be mapped to EORTC QLU‐C10D utility scores.

**Table 4:** Step‐by‐step example: deriving EORTC QLU‐C10D utilities from EORTC QLQ‐C30 data in a prostate cancer trial.

Step	Description
Step 1	Administer EORTC QLQ‐C30 at baseline, 6 months, and 12 months within a prostate cancer RCT
Step 2	Extract 10 EORTC QLQ‐C30 items corresponding to EORTC QLU‐C10D domains: physical functioning, role functioning, social functioning, emotional functioning, pain, fatigue, sleep, appetite loss, nausea, and bowel problems
Step 3	Apply country‐specific DCE‐derived utility weights (e.g., UK value set) to derive a EORTC QLU‐C10D utility score for each patient at each time‐point. Score range: approximately −0.285 to 1.0
Step 4	Calculate area under the utility‐time curve to generate QALYs per patient. Compare between treatment arms to produce an incremental QALY estimate for cost‐utility analysis
Step 5	Compare EORTC QLU‐C10D‐derived QALY estimates against EQ‐5D‐5L results collected in parallel. Differences highlight the incremental sensitivity of the cancer‐specific instrument to prostate cancer treatment effects
Example result	In a prostatectomy vs radiotherapy comparison, EORTC QLQ‐C30‐derived QLU‐C10D scores may detect a −0.08 utility decrement due to fatigue and bowel dysfunction at 6 months after surgery, whereas contemporaneous EQ‐5D scores show only a −0.02 difference – demonstrating the superior responsiveness of the cancer‐specific instrument

EQ‐5D‐5L, EuroQoL five Dimensions five Levels; RCT, randomised controlled trial.

## The Evidence Gap in Non‐Oncological Urology

While cancer‐specific PBMs like the EORTC QLU‐C10D present a significant methodological advance, progress in non‐oncological urology has been more limited. Unlike oncology, where HRQoL and economic evaluation are now more routinely integrated, benign urological conditions remain underrepresented by preference‐based approaches. The lack of validated, urology‐specific PBMs presents a significant barrier to accurate value assessment.

Commonly used PROMs in non‐oncological urology, e.g., the IPSS, OAB‐q, and ICIQ, are not preference based and cannot directly generate utility values. As a result, economic evaluations often rely on generic PBMs or mapping algorithms, each with limitations. Table 5 [23, 24, 25, 26, 27] summarises key trial evidence across this area.

**Table 5:** Key trial evidence: PROMs in economic evaluation of urological conditions.

Trial	Condition	Study comparison	PROM used	QALY result	Key finding
Trans European Research into the use of Management Policies for LUTS suggestive of BPH in Primary Healthcare (TRIUMPH) [24]	Male LUTS	Nurse‐led care vs standard GP management	EQ‐5D‐5L	+0.001 QALYs	Symptoms improved but EQ‐5D failed to detect meaningful HRQoL change – illustrates generic instrument insensitivity in urology
Single‐Incision Mini‐Slings (SIMS) [25]	Stress urinary incontinence	Mini‐sling vs standard mid‐urethral sling	EQ‐5D‐3L	~0.005 QALYs	Negligible QALY differences between mini‐sling and standard sling limited cost‐effectiveness conclusions
Effects of Surgical Treatment Enhanced with Exercise for Mixed Urinary Incontinence (ESTEEM) [26]	Mixed urinary incontinence	Behavioural therapy + surgery vs surgery alone	EQ‐5D	Small gain	Behavioural therapy + sling surgery showed symptom improvement; however, minimal QALY gain and increased cost reduced economic appeal
Refractory Overactive Bladder: Sacral NEuromodulation vs BoTulinum Toxin Assessment (ROSETTA) [27]	Refractory OAB	Sacral neuromodulation vs intradetrusor botulinum toxin A (OnabotulinumtoxinA)	HUI‐3	Equivalent	Sacral neuromodulation vs. Botulinum toxin A: similar utility scores but large cost difference; ICER far exceeded threshold
Chughtai et al. [23]	BPH	Rezum water vapour therapy vs TURP/medical management	IPSS → mapping to utilities	Favoured Rezum	Markov model with IPSS‐mapped utilities showed Rezum offered greater quality‐adjusted life expectancy over 5 years

ICER, incremental cost‐effectiveness ratio; OAB, overactive bladder.

These examples demonstrate a consistent pattern: PROMs are crucial for capturing patient‐centred outcomes, but the tools currently deployed frequently lack sensitivity to clinically meaningful changes in urological health. The TRIUMPH trial (International Standard Randomised Controlled Trial Number [ISRCTN] ISRCTN11669964) is particularly instructive: nurse‐led management improved LUTS symptom scores, yet the EQ‐5D‐5L captured a QALY gain of only 0.001, insufficient to demonstrate cost‐effectiveness under standard NICE thresholds. This illustrates how the choice of HRQoL instrument can effectively determine a study's policy conclusions.

The development of the EORTC QLU‐C10D offers a replicable model for non‐oncological urology. By applying MAUT and DCE methodology to an existing psychometrically validated PROM, researchers produced a tool that is both clinically sensitive and economically useful. Adapting instruments like EPIC‐26 or ICIQ into preference‐based formats would produce urology‐specific PBMs capable of generating utilities directly, improving sensitivity to meaningful changes and reducing reliance on uncertain mapping.

Overall, priorities for non‐oncological urology should include:

- Developing new PBMs specific to benign urological conditions (BPH, LUTS, incontinence, overactive bladder)
- Generating national value sets for these instruments
- Establishing minimally important differences (MIDs) for interpreting utility changes in urological health states
- Expanding use of electronic PROMs (ePROMs) in clinics and registries to support real‐world HRQoL evaluation

Ultimately, this is both a methodological challenge and a matter of equity. Without accurate, preference‐based HRQoL data, the lived experience of patients with urological conditions risks being systematically undervalued in systems increasingly driven by QALY‐based decision‐making.

## Methodological Considerations

The integration of HRQoL and PROMs into economic evaluation presents several methodological challenges that must be addressed to improve the accuracy and relevance of cost‐effectiveness analyses in urology.

### Mapping and its Uncertainty

Many studies rely on mapping disease‐specific PROMs to generic PBMs like the EQ‐5D [28]. While practical, this approach introduces statistical uncertainty and often lacks validation across diverse populations. Direct valuation using preference‐based instruments is preferable but currently limited in urology.

The impact of mapping uncertainty on cost‐effectiveness conclusions can be substantial. Table 6 provides a concrete illustration using a LUTS scenario modelled on the TRIUMPH trial, demonstrating how different mapping functions can shift the incremental cost‐effectiveness ratio (ICER) from well above to well below the NICE threshold of £20 000–30 000/QALY.

**Table 6:** Illustrative impact of mapping uncertainty on ICER – LUTS example.

Scenario	Mapped utility gain	Incremental cost, £	ICER, £/QALY
EQ‐5D directly collected (base case)	+0.001	220	220 000 – above NICE threshold
IPSS mapped to utilities (optimistic mapping)^†^	+0.04	220	5500 – below NICE threshold
IPSS mapped to utilities (pessimistic mapping)^†^	+0.007	220	31 400 – near threshold; conclusion uncertain

ICER, incremental cost‐effectiveness ratio; NICE threshold = £20 000–30 000/QALY.

^†^ Optimistic mapping: uses the upper bound of the 95% CI from the mapping regression, yielding the largest utility gain and lowest ICER. Pessimistic mapping: uses the lower bound of the 95% CI, yielding the smallest utility gain and highest ICER. The base case uses point estimate values directly from EQ‐5D data collection.

This example illustrates that the choice of mapping function, not merely the underlying clinical data, can determine whether an intervention appears cost‐effective. Researchers and HTA reviewers should therefore demand transparency in mapping methodology, including presentation of scenario analyses that reflect the plausible range of mapping assumptions.

### Instrument Sensitivity

Generic PBMs may fail to detect clinically meaningful changes in domains such as urinary function, sexual health, and emotional wellbeing [29]. Instruments tailored to urological conditions are needed to improve sensitivity to treatment effects, as demonstrated consistently across the trials reviewed in Table 5.

### Population‐Specific Utility Weights

Utility weights derived from general population samples may not reflect the preferences of patients living with urological conditions. Developing country‐specific value sets and exploring patient‐derived valuations could enhance relevance [30]. This is particularly pertinent for conditions with significant impact on sexual and urinary function – domains the general population may systematically undervalue relative to affected patients.

### Minimally Important Differences

Establishing MIDs for utility scores is essential for interpreting the clinical significance of changes over time [31]. Without these benchmarks, it is difficult to assess whether observed improvements translate into meaningful patient benefit, or whether small observed QALY differences reflect instrument insensitivity rather than true equivalence.

### Trial Design and PROM Integration

The PROMs must be integrated into clinical trials from the outset, with clear plans for utility derivation [32]. This includes selecting appropriate instruments, ensuring adequate follow‐up, and capturing baseline HRQoL data. Missing data, particularly informative missingness at disease progression or following major complications, represents a further methodological threat that requires prospective planning and appropriate statistical handling.

## Practical Guidance for Researchers and Clinicians

To enhance the integration of HRQoL and PROMs into economic evaluations, researchers and clinicians must adopt a more strategic and collaborative approach. The following guidance reflects the key methodological lessons from this review:

- Select instruments that are both validated and sensitive to urological symptoms, particularly those capable of generating or mapping to utility values. Ideally, studies should incorporate both a generic PBM and a condition‐specific instrument.
- Include a surgery‐related PROM (e.g., CARE) alongside generic instruments in trials evaluating prostatectomy, cystectomy, or other major urological procedures to capture recovery trajectories and perioperative care quality.
- Use ePROM platforms within clinics and registries to improve data completeness and facilitate longitudinal tracking of patient‐reported HRQoL. The ePROMs reduce missing data and support real‐world evaluation.
- Involve health economists from study design inception to ensure PROM data collection is structured to support robust cost‐utility analysis. Early collaboration minimises the need for post hoc mapping and reduces methodological uncertainty.
- When preparing HTA submissions, present both generic and condition‐specific utility estimates alongside sensitivity analyses that account for mapping uncertainty. This dual‐reporting approach supports cross‐disease comparisons while reflecting the full impact of urological interventions.
- Support the adaptation of existing PROMs such as EPIC‐26 and ICIQ into preference‐based formats through collaborative research involving clinicians, methodologists, and patient representatives.

These principles are applicable across the spectrum of urological practice, from trial design in academic centres to outcome reporting in routine clinical services. The integration of these approaches into standard practice represents an achievable and important step toward more equitable and patient‐centred economic evaluation in urology.

## Conclusions

The accurate measurement of HRQoL is critical for valuing urological technologies that are primarily implemented to improve daily functioning rather than extend survival. While oncology has led the development of preference‐based instruments like the EORTC QLU‐C10D, non‐oncological urology remains underserved by such tools.

Current reliance on generic PBMs and mapping approaches risks underestimating the true value of interventions that significantly improve patients’ lives. To address this, the field must develop urology‐specific PBMs, validate mapping algorithms, embed PROMs into both trials and routine care, and integrate surgery‐related instruments (e.g., CARE) into economic evaluations of urological procedures.

This is not merely a methodological refinement; it is a matter of equity and patient‐centred care. Without robust, preference‐based HRQoL data, the lived experience of patients with urological conditions may be systematically undervalued in systems increasingly driven by QALY‐based decision‐making.

A coordinated research agenda, supported by clinicians, researchers, and policymakers, is required to ensure that economic evaluations in urology reflect what matters most to patients.

## Disclosure of Interests

We have no conflicts of interest to declare.

## Data Availability

The data that support the findings of this study are available on request from the corresponding author. The data are not publicly available due to privacy or ethical restrictions.
